# Quality of Life of Women after Giving Birth: Associated Factors Related with the Birth Process

**DOI:** 10.3390/jcm8030324

**Published:** 2019-03-07

**Authors:** Juan Miguel Martínez-Galiano, Antonio Hernández-Martínez, Julián Rodríguez-Almagro, Miguel Delgado-Rodríguez

**Affiliations:** 1 Department of Nursing, University of Jaen, 23071 Jaen, Spain; jgaliano@ujaen.es; 2 Consortium for Biomedical Research in Epidemiology and Public Health (CIBERESP), 28029 Madrid, Spain; mdelgado@ujaen.es; 3 Department of Nursing of University of Castilla la Mancha, 13071 Ciudad Real, Spain; antomatron@gmail.com; 4 Mancha-Centro Hospital, Alcázar de San Juan, 13600 Ciudad Real, Spain; 5 Department of Health Sciences, University of Jaen, 23071 Jaen, Spain

**Keywords:** associated factors, birth, postpartum period, pregnancy, quality of life

## Abstract

The World Health Organization (WHO) considers quality of life a fundamental indicator. The effect of birth on women’s long-term quality of life (QoL) has barely been studied. The purpose of this study was to determine the factors related with the pregnancy, delivery and puerperium, and assess women’s QoL after giving birth. A cross-sectional study with Spanish puerperal women was carried out; it collected data on socio-demographic variables, obstetric variables, newborn data, and several quality of life parameters. An ad hoc online questionnaire, including SF-36 (validated instrument to measure QoL), was used. Crude mean differences (MD) and adjusted mean differences (aMD) were estimated by multiple linear regression. A total of 2990 women participated whose overall QoL lowered with time until three years postpartum (*p* = 0.045). Caesarean section (aMD = −3.61, 95% confidence interval (CI): −5.07, −2.15), mother admitted to ICU (aMD = −4.81, 95% CI: −9.56, −2.68), newborn hospitalized (aMD = −2.10, 95% CI: −4.31, −0.13) or third/fourth degree perineal tears (aMD = −6.87 95%CI: −9.93, −3.82) were detected as risk factors that affect negatively and significantly on postpartum QoL scores. Women’s postpartum QoL worsens with time. Some determining factors negatively influence postpartum QoL: Caesarean section, a mother´s admission to an intensive care unit (ICU) or a premature newborn.

## 1. Introduction

Quality of life (QoL) is a parameter that has been defined and recently taken into account as a health indicator. The World Health Organization (WHO) defines QoL as “the concept of an individual of its own status in life in relation to the culture and the system of values in which it lives, and in relation to its goals, standards and needs” [1].

Several instruments exist to assess QoL in different health and welfare aspects, which include: the SF-36 ((validated instrument to measure QoL) QoL in health questionnaire [2], the EuroQol-5D [3], the King’s Health Questionnaire [4], the QoL questionnaire (WHOQOL-BREF) [5], or the Nottingham Health Profile (NHP) [6], among others. 

The pregnancy, birth and postpartum period, and a child being born along with the new role of mother and the responsibilities that this entails, are significant periods in women’s lives that entail major changes in their QoL due to physical, psychic and social repercussions [7,8,9].

Different aspects of the pregnancy, birth and postpartum process and QoL have been studied [10,11,12,13,14,15,16] but, as a literature review of 75 studies indicates, very few research works have evaluated QoL during pregnancy [14]. An Australian cohort study detected how the physical QoL of 355 women significantly worsened throughout pregnancy, in contrast mental QoL tended to improve, but not significantly so [10]. A Swedish study followed up a sample of 372 primiparous women during five years postpartum. This research related birth with the QoL score and some of its dimensions. Some of those worsened, such as their former health status, how they generally perceived their health, their disease resistance and also their health concerns [11]. For the 190 Japanese women who participated in a study into QoL during pregnancy, the subscale scores for “physical function”, “physical role” and “bodily pain” significantly decreased throughout pregnancy, while no significant changes during pregnancy were noted for the “general health” and “mental health” subscales [15].

Given the importance that QoL has during the pregnancy, birth and postpartum process, and the recommendations made for future studies into this topic to design suitable strategies, policies and programmes to improve women’s postpartum QoL, the aim to determine the factors related with the pregnancy, birth and postpartum process associated with postpartum QoL was proposed.

## 2. Materials and Methods

A cross-sectional study was conducted with women who gave birth in Spain in 2017.

This study was approved by the Ethics Committee in Clinical research (CEIC) of Mancha-Centro Hospital. Before the women completed the study questionnaire, they were given information about the study, its objectives, etc. They ticked a box if they wished to give their consent; i.e., they signed an ad hoc devised digital informed consent.

Births that resulted in antenatal fetal death and women aged under 18 years old were excluded. 

A 5% alpha risk, a 10% beta risk (power = 90%), an effect size of 0.02 points and a potential number of 10 predictors were applied to estimate sample size [17]. The minimum sample size was estimated at 1036 women. 

### 2.1. Sources of Information

The authors designed an online questionnaire for data collection. It included 35 items (3 open, 32 closed questions) about obstetric results, clinical and socio-demographic characteristics, and newborn data. The validated instrument SF-36 [2,18] was also used to measure QoL. The questionnaire had been formerly piloted and handed to women by the main women’s associations, the Spanish Federation of Midwives Associations (FAME). In order to encourage women´s participation its member associations and their midwives made the project known and helped facilitate it. Having selecting the subjects and having them sign the consent to participate, they received instructions to complete the questionnaire (self-completed), which they did when appropriate. A phone number and a chat service were used to answer any queries they had when answering the questionnaire.

The following variables were collected:

Our main dependent variable was the score obtained with the SF-36 QoL questionnaire.

The independent variables were: the mother’s age, educational level, nationality, parity (primiparous or multiparous), health problems during pregnancy, previous caesarean section, twin pregnancy, birth type (vaginal or caesarean section), third/fourth degree perineal tears, involved episiotomy during birth, mother admitted to an intensive care unit (ICU) after giving birth, postpartum surgery, mother readmitted to hospital after being discharged, premature newborn, newborn hospitalized, breastfeeding, postpartum time (less than 1 year, 1–3 years, more than 3 years) and if the women’s QoL was affected by different processes, except pregnancy, birth and postpartum. For this variable, we created a specific question/item in the questionnaire. The woman was asked what her perception was about whether or not it affected her socio-economic level, the presence of pathology or other factors that were not related to the pregnancy process on her quality of life.

### 2.2. Statistical Analysis Employed

First of all, a descriptive analysis was run with absolute and relative frequencies for the categorical variables, and the mean with standard deviation (SD) for the quantitative variables. An analysis of variance (ANOVA) was used to compare the QoL scores according to their last postpartum time. To establish any relationship among the different factors and QoL, the crude mean difference (MD) of the scores was calculated by linear regression. Then the adjusted MD (aMD) was calculated by multiple linear regression following the SPSS forward and backward procedures by introducing the potential confounder variables into the analysis. Finally, for the estimation of the individual quality of life a simple calculator was developed with the Excel program. The final score was calculated by means of a linear regression equation. In this resource, the variables that were detected that had an association with the quality of life were included (Supplementary Table S1).

A *p* ˂ 0.05 was considered significant. All the analyses were performed with the SPSS v24.0 (SPSS Inc., Chicago, IL, USA) statistics package.

### 2.3. Ethical Approval

This study was approved by the Ethics Committee in Clinical Research (CEIC) of the Mancha-Centre Hospital with reference number ACT 2017.

## 3. Results

In this work, 2990 women participated: 59.1% (1767) had studied at university, the pregnancies of 70.7% (2113) ended normally, 78.5% (2257) had a normal vaginal birth, for 63.6% (1901) no episiotomy was involved, 70.6% (2741) breastfed their infants and 66.5% (1987) of the studied cases had spent more than one year during the intergenesic period, as Table 1 shows with its study population characteristics.

Table 2 offers the overall QoL scores and the specific dimensions that form it at three specific time points: Before 1 year postpartum, 1–3 years postpartum and more than 3 years postpartum. We can see how QoL tends to worsen with time, as the overall QoL score indicates (*p* = 0.045), and also some of its dimensions, like overall health (*p* < 0.001), mental health (*p* = 0.005), etc.

Among the other variables in Table 3, we find having completed secondary (aMD = 14.10, 95% CI: 1.55, 26.65) and university (aMD = 18.70, 95% CI: 6.15, 31.25) education helped to obtain a higher postpartum QoL score. Caesarean section (aMD = −3.61, 95% CI: −5.07, −2.15), mother admitted to an ICU (aMD = −4.81, 95% CI: −9.56, −2.68), newborn hospitalized (aMD = −2.10, 95% CI: 4.31, −0.13), third/fourth degree perineal tears (aMD = −6.87, 95% CI: −9.93, −3.82) or involved episiotomy (aMD = −4.81, 95% CI: −9.56, −2.68) while giving birth were risk factors that significantly and negatively affected the postpartum QoL score.

As an example of how the resource that has been created to calculate the quality of life of women in the postpartum period would work: The case of a woman over 35 years old, with secondary studies, multipara, with problems during pregnancy, with a cesarean delivery, without perineal tears type III/IV, without admission to the ICU, without hospital admission after discharge, with admission of the newborn, without affecting their quality of life due to other problems other than pregnancy, childbirth and puerperium would have to solve the following equation and that its delivery occurred between 1 and 3 years. 

**Figure 1:**
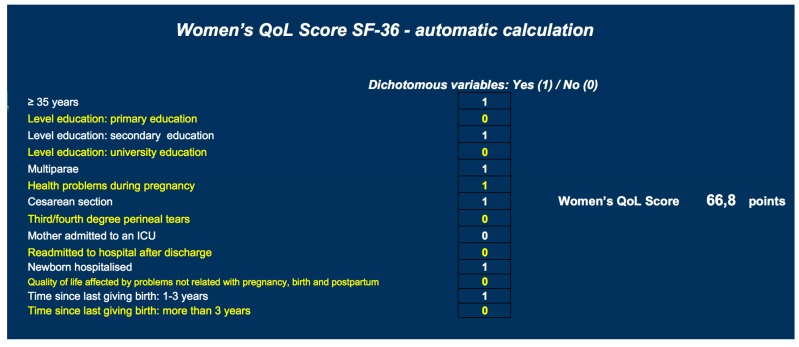
Score obtained in the SF-36 of the example created to explain the operation of the calculator.

Score SF-36 = (Cte = 58.69) + ((age ≥ 35 years = 1) × − 1.24) + ((level education secondary = 1) × 14.10) + ((multipara = 1) × 1.77) + ((health problems during pregnancy = 1) × −2.42) + ((caesarean section = 1) × −3.61) + ((third/fourth degree perineal tears = 0) × − 6.87) + ((mother admitted to an ICU = 0) × − 4.81) + ((readmitted to hospital after discharge = 0) × − 6.12) + ((newborn hospitalized = 1) × − 2.10) + ((Quality of life affected by problems not related with pregnancy, birth and postpartum = 0) × − 13.52) + ((time since last giving birth: 1–3 years = 1) × 1.64). Score SF-36 = 66.8 points. (Figure 1).

## 4. Discussion

The overall QoL score of those women affected by maternity lowered with time. Some dimensions at <1 year postpartum, e.g., the “emotional role”, scored well, but then significantly lowered at 1–3 years to then increase after three years postpartum. Some dimensions, e.g., the vitality score, barely changed, while others, e.g., the “physical role”, obtained higher scores at 1–3 years postpartum, before dropping at three years, but were higher than during the initial postpartum period. Having had a higher education level was identified as a factor that facilitated women obtaining a higher postpartum QoL score. Caesarean section, third/fourth degree perineal tears while giving birth, involved episiotomy, having a premature newborn, the mother being admitted to an ICU, hospital readmission or the newborn being hospitalized were factors associated with women obtaining a lower postpartum QoL score. 

A priori, the use of the calculator is designed for the reference population. However, it can be useful at a global level, as it provides the basis for a study that can be carried out in other populations. In this way, it would be possible to include in the resource those variables suitable for each population, as occurs with a very generalized practice: the validation of questionnaires. In addition, the obtained results could be compared between different populations.

No information was collected about depression during pregnancy and/or postpartum. The presence of this pathology can have an important effect on the quality of life of women in the postpartum period but in our study we could not associate it because this information was not available. The association of factors unrelated to the process of pregnancy and childbirth such as, economic level, etc., with the quality of life was determined independently with a specific question. This variable was used to adjust the score in the multivariate model. It is evident that the influence of the quality of life that a woman has, without taking into account the factors of the process of pregnancy and childbirth, can determine significantly the quality of life of the woman in the postpartum period. To avoid confusion of this variable on our results, we decided to adjust for it.

If there was a selection bias associated with non-response, it did not influence the results. The sample is representative of the Spanish population. Therefore, we think that women who did not respond, a priori, there are no reasons to think that they had done it in a different way to those who did respond. No information bias is likely to exist: the data collected combined with the questionnaire setup did not require a high educational level as was simply described. The data collection was carried out in a short time interval, we consider that the possible forgetfulness would have minimally affected the results because women perfectly know and remember the information about their birth process.

This study has its strong points: a large sample size, women from different geographical areas and is representative of the Spanish population. The instrument we used was the validated SF-36 questionnaire [2] as it has been used with pregnant Spanish women [19]. 

Most previous studies [19,20,21,22,23,24,25,26,27,28] refer to QoL during pregnancy and a short postpartum period, e.g., six weeks or six months, but have not dealt with long-term QoL as our results do, which measure QoL up to three years postpartum. Our results overall indicate QoL loss during the 1–3 year postpartum period compared to the first postpartum year. The score lowered again at three years postpartum. This poorer QoL falls in line with what Parks and Choi detected in their study conducted in South Korea with 5146 women aged more than 50 years. These authors identified an association of different parameters related to the pregnancy, birth and postpartum process and QoL [29]. The “physical role” dimension increased the score as the postpartum time prolonged (1–3 years), but it lowered at three years postpartum. All the dimensions lowered the score with time, but only significantly so for general health, the emotional role and mental health.

The mother’s age ≥35 years did not come over as a QoL-related factor, which contrasts with what Parks and Choi found because they identified the mother’s younger age at child birth as a determining factor for worse QoL [29]. Another study included three clinical trials (DIGITAT, HYPITAT and WOMB) and did not associate the mother’s age with QoL [27], which is in line with our results.

Among the factors identified as being predisposed to better QoL during the postpartum period, our results associated with women having secondary or university educations as opposed to those with no higher education. Yet other authors have found no such association [27]. This could perhaps be explained by having higher education implying better self-perceived health [30]. We found no association between women’s nationality and postpartum QoL, as other authors have done [27].

The parity reflected in our results was associated with postpartum QoL, unlike what Prick et al. reported [27], that parity had no impact on postpartum QoL, but discussed by other authors [20,21,29] as they detected this association. However, Bai et al. [21] found that nulliparity negatively affected postpartum QoL, while Park and Choi [29] stated that multiparity was a determining factor for lower QoL.

Having health problems during pregnancy, like high blood pressure, nausea, anxiety and gestational diabetes, was another predisposing factor for worse postpartum QoL in our study, which coincides with other research results [20,21,27]. Caesarean section was also associated with worse postpartum QoL, which agrees with other works [27]. Nonetheless, Triviño-Juárez et al. [19] did not identify birth type as a factor that affected postpartum QoL in their Spanish study conducted with 546 women.

We observed how third/fourth degree perineal tears and involved episiotomy were associated with worse postpartum QoL, while others found no such relation [27]. However, their studies did not distinguish among different perineal lesion types, which our study did, but considered more severe perineal lesions that cause greater discomfort [31,32].

Similar to Prick et al. [27], newborn hospitalization and the mother being readmitted to hospital after being discharged, which falls in line with Fobelets et al. [26], and the mother being admitted to an ICU, which agrees with Pia et al. [24], were identified in our results as determining factors of worse postpartum QoL. The outcomes reported by Seppänen et al. [25] informed that the QoL of the women admitted to an ICU at six months postpartum tended to be similar scores to those given by the general population.

The gestational age at which women gave birth was also identified as a risk factor for a worse QoL score, which agrees with other studies [22,33].

The way babies were fed was studied. The women with formula-fed babies did not obtain worse QoL scores than those who breast-fed, which contrasts with what Triviño-Juárez et al. [28] identified as these authors associated breast feeding with a better QoL at six weeks postpartum.

Having a poor QoL before pregnancy due to factors unrelated to the pregnancy, birth and postpartum process was also identified as a predisposing factor for low postpartum QoL scores, which coincides with Fobelet et al. [26]. The women with a low QoL before being pregnant, for non-related to pregnancy reasons, birth and postpartum period, could be affected by this process because it is a stressful situation [20] that could affect negatively to further worsen or aggravate their QoL.

In Table S1 of the Supplemental Material, there is a calculator that can be used by professionals to include required information and individually calculate women’s QoL scores, which is useful for identifying risk populations.

## 5. Conclusions

As a conclusion, postpartum women obtain QoL scores that lower with postpartum time. Certain factors are related with the pregnancy, birth and postpartum period, such as type of birth, a pre-term newborn, health problems during pregnancy, etc., that negatively impact women’s QoL. Healthcare policies and perinatal health programs must bear these factors in mind to set up measures in order to prevent and improve women’s postpartum QoL.

## Figures and Tables

**Table 1:** Characteristics of the study population.

Variable	*n* (%)
Mother’s age	
<35 years	1449 (48.5)
≥35 years	1541 (51.5)
Educational level	
No studies	7 (0.2)
Primary education	146 (4.9)
Secondary education	1070 (35.8)
University education	1767 (59.1)
Nationality	
Spanish	2886 (96.5)
Other	104 (3.5)
Parity	
Primiparae	1503 (50.3)
Multiparae	1487 (49.7)
Health problems during pregnancy	
No	2113 (70.7)
Yes	877 (29.3)
Previous Caesarean section	
No	2072 (69.3)
Yes	918 (30.7)
Twin pregnancy	
No	2870 (96.0)
Yes	120 (4.0)
Type of Birth	
Vaginal	2257 (75.5)
Caesarean section	733 (24.5)
Episiotomy	
No	1901 (63.6)
Yes	1089 (36.4)
Third/fourth degree perineal tears	
No	2865 (95.8)
Yes	125 (4.2)
Mother admitted to an ICU	
No	2928 (97.9)
Yes	62 (2.1)
Performing postpartum surgery	
No	2921 (97.7)
Yes	69 (2.3)
Readmitted to hospital after discharge	
No	2893 (96.8)
Yes	97 (3.2)
Premature newborn	
No	2757 (92.2)
Yes	233 (7.8)
Newborn hospitalised	
No	2741 (70.6)
Yes	249 (8.3)
Formula-fed	
No	2112 (70.6)
Yes	878 (29.4)
Time since last giving birth	
Less than 1 year	1003 (33.5)
1–3 years	1091 (36.5)
More than 3 years	896 (30.0)

**Table 2:** Overall scores for quality of life and dimensions according to time points.

Variable	Time since Last Birth			*p*-Value *	*p*-Value * for Linear Tendency
	Less than 1 Year Mean (SD)	between 1–3 Years Mean (SD)	More than 3 Years Mean (SD)		
Overall quality of life	71.94 (17.48)	71.75 (18.01)	70.23 (19.25)	0.084	0.045
Physical function	89.46 (20.72)	91.27 (18.76)	89.11 (22.58)	0.040	0.786
Physical role	65.25 (44.52)	72.96 (40,78)	70.53 (42.04)	<0.001	0.005
Bodily pain	85.34 (22.77)	84.99 (23.87)	83.40 (24.67)	0.168	0.079
General health	63.11 (18.76)	60.01 (19.89)	57.28 (19.72)	<0.001	<0.001
Vitality	51.72 (23.00)	51.27 (22.39)	51.23 (23.66)	0.868	0.632
Social role functioning	75.70 (25.99)	74.68 (26.98)	74.41 (26.08)	0.525	0.284
Emotional role	80.61 (36.98)	76.58 (39.83)	73.88 (41.53)	0.001	<0.001
Mental health	64.31 (18.08)	62.23 (18.58)	61.96 (18.97)	0.009	0.005

* Analysis of variance; SD, standard deviation.

**Table 3:** Factors associated with postpartum quality of life.

Variable	Quality of Life Scores Mean (SD)	MD 95%CI *	aMD 95%CI **
Mother’s age			
<35 years	71.84 (18.04)	(ref.)	(ref.)
≥35 years	70.90 (18.39)	−0.93 (−2.24, 0.37)	−1.24 (−2.55, 0.08)
Level of education			
No studies	60.09 (23.27)	(ref.)	(ref.)
Primary studies	66.62 (19.94)	6.53 (−7.19, 20.25)	12.42 (−0.39, 25.24)
Secondary studies	68.94 (19.61)	8.85 (−4.59, 22.30)	**14.10 (1.55, 26.65)**
University studies	73.26 (16.90)	13.17 (−0.26, 26.59)	**18.70 (6.15, 31.25)**
Nationality			
Spanish	71.48 (18.16)	(ref.)	
Not Spanish	67.93 (19.60)	−3.55 (−7.12, 0.01)	
Parity			
Primiparae	70.25 (18.54)	(ref.)	(ref.)
Multiparae	72.48 (17.84)	2.23 (0.93, 3.54)	**1.77 (0.53, 3.02)**
Health problems during pregnancy			
No	71.84 (18.60)	(ref.)	(ref.)
Yes	68.19 (18.36)	**−3.65 (−5.57, −1.73)**	**−2.42 (−4.22, −0.63)**
Previous caesarean section			
No	72.41 (17.82)	(ref.)	
Yes	68.98 (18.90)	**−3.44 (−3.85, −2.03)**	
Twin pregnancy			
No	71.40 (18.18)	(ref.)	
Yes	70.28 (19.16)	−1.13 (−4.46, 2.20)	
Birth type			
Vaginal	72.46 (17.77)	(ref.)	(ref.)
Caesarean section	67.95 (19.19)	**−4.51 (−6.02, −2.99)**	**−3.61 (−5.07, −2.15)**
Third/fourth degree perineal tears			
No	71.66 (18.07)	(ref.)	(ref.)
Yes	64.46 (20.29)	**−7.20 (−10.45, −3.94)**	**−6.87 (−9.93, −3.82)**
Episiotomy			
No	71.32 (18.26)	(ref.)	
Yes	71.42 (18.18)	0.10 (−1.26, 1.46)	
Mother admitted to an ICU			
No	71.54 (18.08)	(ref.)	(ref.)
Yes	62.49 (22.53)	**−9.06 (−13.63, −4.48)**	**−4.81 (−9.56, −2.68)**
Performing postpartum surgery			
No	71.49 (18.17)	(ref.)	
Yes	65.75 (19.79)	**−5.74 (−10.09, −1.39)**	
Readmitted to hospital after discharge			
No	71.57 (18.08)	(ref.)	(ref.)
Yes	65.07 (21.30)	**−6.50 (−10.18, −2.82)**	**−6.12 (−9.14, −0.47)**
Premature newborn			
No	71.66 (18.07)	(ref.)	
Yes	67.74 (19.65)	**−3.92 (−6.35, −1.48)**	
Newborn hospitalized			
No	71.65 (17.96)	(ref.)	(ref.)
Yes	68.10 (20.66)	**−3.55 (−5.92, −1.19)**	**−2.10 (−4.31, −0.13)**
Formula-fed			
No	72.26 (17.48)	(ref.)	
Yes	69.19 (19.75)	**−3.07 (−4.50, −1.64)**	
Time since last given birth			
Less than 1 year	71.94 (17.48)	1 (ref.)	(ref.)
1–3 years	71.75 (18.01)	−0.19 (−1.75, 1.37)	**1.65 (0.18, 3.11)**
More than 3 years	70.23 (19.25)	**−1.71 (−3.35, −0.06)**	1.20 (−0.42, 2.81)
Quality of life affected by problems not related with pregnancy, birth and postpartum			
No	74.67 (16.64)	1 (ref.)	(ref.)
Yes	60.99 (19.08)	**−13.69 (−15.13, −12.24)**	**−13.52 (−14.94, −12.09)**

* MD: Crude mean differences. ** aMD: Mean differences adjusted by level of education, mother’s age, parity, problems during pregnancy, birth type, third/fourth degree perineal tears, mother admitted to an ICU, postpartum surgery, mother readmitted to hospital after discharge, newborn hospitalized, intergenesic period, quality of life affected by problems unrelated to pregnancy, birth and postpartum; (ref.): reference.

## Supplementary Materials

The following are available online at https://www.mdpi.com/2077-0383/8/3/324/s1, Table S1: Instrument to automatic calculation of Women’s QoL Score SF-36.
